# Interaction of Hyperalgesia and Sensory Loss in Complex Regional Pain Syndrome Type I (CRPS I)

**DOI:** 10.1371/journal.pone.0002742

**Published:** 2008-07-23

**Authors:** Volker Huge, Meike Lauchart, Stefanie Förderreuther, Wibke Kaufhold, Michael Valet, Shahnaz Christina Azad, Antje Beyer, Walter Magerl

**Affiliations:** 1 Department of Anaesthesiology, Ludwig-Maximilians-Universität München, Munich, Germany; 2 Department of Neurology, Ludwig-Maximilians-Universität München, Munich, Germany; 3 Department of Neurology, Technische Universität München, Munich, Germany; 4 Center of Biomedicine and Medical Technology (CBTM), Department of Neurobiology, Medical Faculty Mannheim, Ruprecht-Karls-Universität Heidelberg, Mannheim, Germany; 5 Department of Physiology and Pathophysiology, Johannes Gutenberg Universität Mainz, Mainz, Germany; Women's and Children's Hospital, Australia

## Abstract

**Background:**

Sensory abnormalities are a key feature of Complex Regional Pain Syndrome (CRPS). In order to characterise these changes in patients suffering from acute or chronic CRPS I, we used Quantitative Sensory Testing (QST) in comparison to an age and gender matched control group.

**Methods:**

61 patients presenting with CRPS I of the upper extremity and 56 healthy subjects were prospectively assessed using QST. The patients' warm and cold detection thresholds (WDT; CDT), the heat and cold pain thresholds (HPT; CPT) and the occurrence of paradoxical heat sensation (PHS) were observed.

**Results:**

In acute CRPS I, patients showed warm and cold hyperalgesia, indicated by significant changes in HPT and CPT. WDT and CDT were significantly increased as well, indicating warm and cold hypoaesthesia. In chronic CRPS, thermal hyperalgesia declined, but CDT as well as WDT further deteriorated. Solely patients with acute CRPS displayed PHS. To a minor degree, all QST changes were also present on the contralateral limb.

**Conclusions:**

We propose three pathomechanisms of CRPS I, which follow a distinct time course: Thermal hyperalgesia, observed in acute CRPS, indicates an ongoing aseptic peripheral inflammation. Thermal hypoaesthesia, as detected in acute and chronic CRPS, signals a degeneration of A-delta and C-fibres, which further deteriorates in chronic CRPS. PHS in acute CRPS I indicates that both inflammation and degeneration are present, whilst in chronic CRPS I, the pathomechanism of degeneration dominates, signalled by the absence of PHS. The contralateral changes observed strongly suggest the involvement of the central nervous system.

## Introduction

Complex Regional Pain Syndrome (CRPS) is a neuropathic pain disorder, evolving after limb trauma either without (CRPS I), or with definable nerve lesion (CRPS II) [1]. Beyond pain, autonomic, trophic and motor disturbances, sensory abnormalities are key symptoms of CRPS [2], typically not confined to the innervation territory of peripheral nerves or nerve roots. Conspicuously, CRPS sensory abnormalities may spread in a hemisensory manner [3] or even contralaterally [4]. No generally accepted animal model of CRPS I exists, but very recently an ischemia-reperfusion injury model reproduced some changes observed in humans [5] (but see [6]).

A sequence of disease symptoms has been described [7], [8], characterized by initial signs of regional inflammation (edema and sudomotor disturbances), followed by functional atrophy. Others identified three distinct subtypes, but debated a chronological succession [9].

Several hypotheses about the pathophysiology of CRPS have been proposed emphasizing the importance of peripheral neurogenic inflammation [10], [11], small-fibre axonal degeneration [12], [13] or central changes (cortical reorganisation) [14]–[16] similar to other pain disorders [17], since cortical changes were related to measures of pain plasticity (hyperalgesia) rather than spontaneous pain [14], [15]. Others suggested an interaction of peripheral and central nervous changes [18].

Although sensory alterations accompanying CRPS can be assessed by quantitative sensory testing (QST) [19], little is known about the distinct patterns of these changes, nor their possible time course. A standardised controlled prospective analysis of the sensory changes in acute and chronic stages of CRPS I might provide novel insights into the pathophysiology of the disease. Moreover, since the majority of sensory data rely on side-to-side comparison [3], [19], a rigorous investigation into the possible presence and magnitude of contralateral symptoms is of major importance.

Therefore, we conducted a prospective study, investigating an unbiased sample of patients suffering from acute or chronic CRPS I of the upper extremity as well as an age- and gender-matched healthy control group. Patients with CRPS II or CRPS of the lower extremities were excluded, since the probable pathophysiology of CRPS I differs from CRPS II, and sensory thresholds of upper and lower extremities differ significantly [20]. The goal of the study was to delineate differences in the ipsi- and contralateral sensory profiles in acute and chronic CRPS I.

## Material and Methods

### Patients and Control Subjects

**Figure 1:**
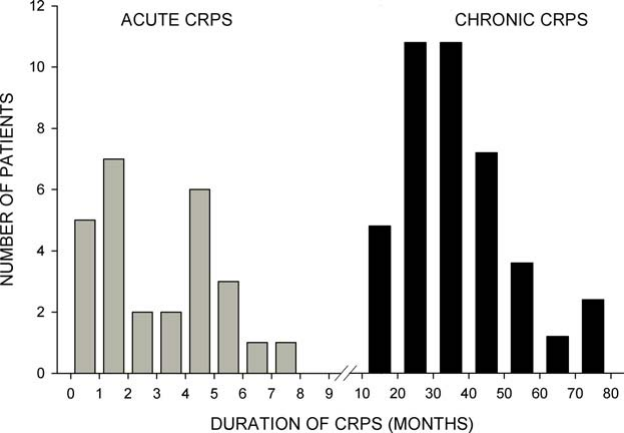
Duration of disease in patients with acute and chronic CRPS. No intersection between both groups occurred.

Over a period of nine months, 61 consecutive patients presenting with the diagnosis CRPS I of the upper extremity (7 male, 54 female, mean age 59.1±12.9 years) agreed to participate in the study. The diagnosis CRPS I was established by experienced examiners according to the research diagnosis criteria proposed by Bruehl [2] (Table 1) as well as the IASP criteria for CRPS [1]. Duration of CRPS was defined as the time since the inciting event, as the beginning of CRPS symptoms could not always be clearly defined. Patients assigned into two groups related to duration of disease: patients with CRPS for twelve months or less were considered as “acute CRPS” (n = 27), while patients with a longer history (>12 months, n = 34) were considered as “chronic CRPS” (Figure 1). This cut-off point was chosen in accordance with the stages as described by Bonica [8]. 56 healthy subjects, matched for gender and age (16 males, 40 females, mean age 56.8±12.3 years) were examined in the same way as the CRPS patients. Demographic data of patients and control subjects are shown in table 2. All patients and subjects presenting with diseases other than CRPS, which potentially affect sensory testing, i.e. diabetes, polyneuropathy, as well as individuals with mental disease were excluded from the study. Also, patients with hearing or speech disorders, as well as patients with other communication problems were excluded. The study was approved by the local ethics committee, and written informed consent was obtained by all subjects enrolled in the study according to the Declaration of Helsinki.

**Table 1:** Proposed modified research diagnostic criteria for CRPS. *

*1: Continuing pain which is disproportionate to any inciting event*	
2: Must report at least one symptom in each of the four following categories	3: Must display at least one sign in two or more of the following categories
**Sensory:** reports of hyperesthesia	**Sensory:** evidence of hyperalgesia (to pinprick) and/or allodynia (to light touch)
**Vasomotor:** reports of temperature asymmetry and/or skin color changes and/or skin color asymmetry	**Vasomotor:** evidence of temperature asymmetry and/or skin color changes and /or asymmetry
**Sudomotor/edema:** reports of edema and/or sweating changes and/or sweating asymmetry	**Sudomotor/edema:** evidence of edema and/or sweating changes and/or sweating asymmetry
**Motor/trophic:** reports of decreased range of motion and/or motor dysfunction (weakness, tremor, dystonia) and or throphic changes (hair, nail, skin)	**Motor/trophic:** evidence of decreased range of motion and/or motor dysfunction (weakness, tremor, dystonia, and/or trophy changes (hair, nail, skin)

* Bruehl S, Harden RN, Galer BS et al. External validation of IASP diagnostic criteria for Complex Regional Pain Syndrome and proposed research diagnostic criteria. International Association for the Study of Pain. Pain 1999; 81: 147–154.

**Table 2:** Characteristics of CRPS patients and control subjects.

	Control (n = 56)	All CRPS (n = 61)	Acute CRPS (n = 27)	Chronic CRPS (n = 34)	P-value CRPS vs. Controls	P-value Acute vs. Chronic CRPS
Age (years) Mean±SD	56.8±12.3	59.1±12.9	56.0±12.8	61.6±12.5	0.327 *	0.092 *
Gender (female/male)	40 / 14	54 / 7	25 / 2	29 / 5	0.078 #	0.628 #
Time since inciting event (months) Mean±SD		22.4±20.4	3.5±2.1	37.4±15.1		<0.0001 *
Pain rating (NRS) Mean±SD		2.48±2.62 (n = 60)	2.77±2.55 (n = 26)	2.26±2.69		0.853*
Presence of Edema (%)		31 / 59 (52.5%	24 / 25 (96.0%)	7 / 34 (20.6 %)		<0.0001#
Difference in skin temperature (Ipsi- vs. contralateral hand) Mean±SD		−0.03±0.89 (n = 58)	+0.25±1.00 (n = 24)	−0.22±0.78		<0.05*
Depression Score (CES-D) Mean±SD		18.7±10.2 (n = 45)	19.5±9.5 (n = 21)	18±10.9 (n = 24)		0.612*

* unpaired t-test

# Yates-corrected chi-square

### Testing Conditions

All tests were performed under minimal distraction in a silent, air-conditioned room, with an ambient temperature of 25–26°Celsius. Subjects were seated on a comfortable chair, and allowed to adapt to the test environment for at least 20 minutes. QST followed a standardised protocol as described by Rolke and colleagues [21], but solely the thermal testing part of this protocol was performed. The course of assessments was explained to the subjects by written standard patient instructions. All sensory tests were demonstrated in a remote test area (forearm) not affected by the underlying disease.

### Equipment

Thermal testing was performed using a Medoc Thermal Stimulus Analyser TSA-2001 device (Medoc, Ramat Yishai, Israel) using a computer-controlled Peltier-based probe. The basic principles of the Peltier stimulator are described in detail elsewhere [22]. The 30×30 mm Peltier element was attached to the patients' hand dorsum by means of an elastic tape. The probe was placed in a way that an optimal contact between the hand and the probe was achieved. The hand affected by the disease was termed “ipsilateral”, while the other hand was termed “contralateral”. Thermal testing commenced in the contralateral side. In healthy control subjects, the dominant hand was termed “ipsilateral”.

### Test Algorithm

Warm detection threshold (WDT), cold detection threshold (CDT), thermal sensory limen (TSL), cold pain threshold (CPT), and heat pain threshold (HPT) were measured using the method of limits as follows: For determination of WDT, CDT, CPT and HPT, subjects were applied three successive stimulations starting from a baseline temperature of 32°C. The rate of temperature increase or decrease respectively was 1°C/s. For WDT and CDT, subjects were instructed to press the response button as soon as sensation of cold or warm was detected. For detection of CPT and HPT the patients pressed the button to indicate the onset of cold pain or heat pain. After the button was pressed, the temperature returned immediately to baseline. For safety reasons, the minimal and maximal temperatures allowed were 0°C and 50°C to avoid tissue damage. If the individual pain threshold was not reached within these confines, 0°C or 50°C were assigned as surrogate pain threshold measures. A computer-generated random interval ranging from five to fifteen seconds was intercalated between stimulations. TSL was estimated as the difference limen for cold and warm thresholds when cold and warm stimuli were given in alternating order. For this reason, six alternating warm and cold stimuli were applied without returning to the baseline temperature. Patients were instructed to press the test button when a warm or cold sensation was felt. Subjects were also asked to identify the quality of the sensation at any time a cold or warm stimulus was given. Identification of a cold stimulus as either hot or burning pain was denoted as the occurrence of paradoxical heat sensation (PHS) [23]. For additional details see Rolke and colleagues [21]. The standard order of tests for all patients was: CDT, WDT, TSL, CPT, and HPT. Total duration of sensory testing was about 45 minutes.

### Clinical Presentation

In patients presenting with CRPS, clinical data were assessed with a standardised protocol obtaining data on the magnitude of pain, edema, skin temperature and levels of depression. Pain ratings were assessed by means of a numeric rating scale (NRS) from 0 to 10. Patients reported the pain level in the ipsilateral hand at the time of presentation. Furthermore, the presence or absence of edema was evaluated by the examiner in a dichotomous way (yes/no). Skin temperature was measured in three consecutive tests on the dorsum of each hand, applying infrared thermometry (PROSCAN 510, TFA Dostmann, Reicholzheim, Germany). Symptoms of depression were assessed by means of the German version of the *Center for Epidemiological Studies Depression Test* (CES-D). This test combines twenty questions designed to measure levels of depression [24], [25]. A raw test score of 27 or more is considered to be the critical limit for the presence of a depressive episode in pain patients [26].

### Data Analysis

Demographic and clinical data were compared using an unpaired t-test. Gender proportions as well as the presence of edema were tested by Yates-corrected chi-square. Kolmogorov-Smirnov's test was performed to assess deviations from normal distribution in demographic data.

For analysis of WDT and CDT, the mean of the three measurements of temperature change from the baseline temperature of 32°C was calculated. Likewise, TSL was calculated as the mean difference between cold and warm thresholds at alternating stimulation. CDT, WDT and TSL data were transformed into decadic logarithms to achieve secondary normal distributions of these data. For CPT and HPT the arithmetic means were used for analysis. Data were analysed by two way analysis of covariance (ANCOVA) with the main factors: group (controls, acute and chronic CRPS) and body side (ipsilateral vs. contralateral), and the covariate age in order to control for the known age-dependency of somatosensation [20]. The locus of significance was identified by post hoc least significant differences (LSD) tests.

Data were also normalized relative to the mean and standard deviation (SD) of the control group (z-transformation) according to the formula: z = (x−mean_control_)/SD_control_. This operation rendered all z-transformed data directly comparable in units of SD of the control group. Z-transformed data were analysed by four way analysis of covariance (ANCOVA) with the main factors: group (controls, acute and chronic CRPS), body side (ipsilateral vs. contralateral), sensory dimension (nociceptive vs. non-nociceptive), and thermal modality (hot vs. cold) and the covariate age.

The impact of the presence or absence of PHS was also analyzed by two way analysis of covariance (ANCOVA) with the main factors: PHS (PHS+ vs. PHS−) and body side (ipsilateral vs. contralateral), and the covariate age. In addition, all parameters were entered into a forward stepwise multiple regression equation, in order to identify which parameters significantly predicted the occurrence of PHS. A probability level of p<0.05 was considered significant, p<0.10 was considered a significant trend. All analysis was performed using the STATISTICA^®^ software package (STATISTICA^®^ 4.5, Statsoft, Tulsa, USA).

## Results

### Patients and Control Subjects

There was no difference in mean age between CRPS I patients and healthy subjects, both groups exhibited normal distributions of age. CRPS duration, however, exhibited an obvious bimodal distribution (Figure 1, Table 2).

No differences were found concerning the levels of pain as well as depression between acute and chronic CRPS patients (Table 2). However, almost all patients in the acute CRPS group displayed clinical signs of edema (24/25 patients = 96%), which were only present in a minority of chronic CRPS patients (7/34 patients = 20.6%, p<0.0001; Table 2). Overall, there was no difference in skin temperature between both hands. However, in the acute CRPS group, the ipsilateral hand was warmer when compared to the contralateral hand (Δ = +0.25±1.00°C), whilst in the chronic CRPS group, the ipsilateral hand was colder (Δ = −0.22±0.78°C). When comparing both groups, this difference reached statistical significance (p<0.05).

### Thermal Detection Thresholds

**Figure 2:**
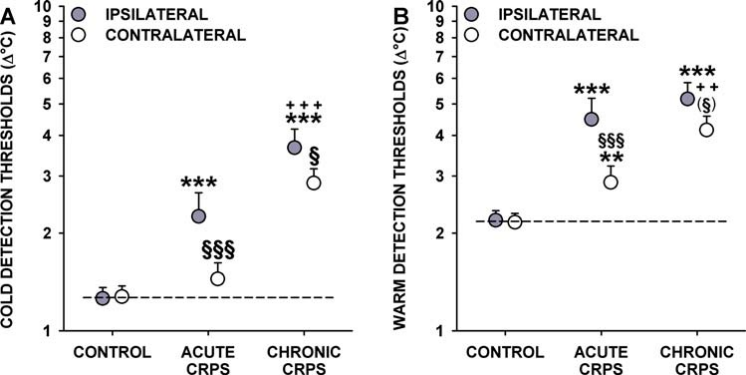
Thermal detection in acute and chronic CRPS. Cold detection thresholds (A) and warm detection thresholds (B), shown as change (Δ°C) from the adaption temperature of 32°C. Thresholds are significantly increased in acute and chronic CRPS. Significant increases are also found in the contralateral “unaffected” hand. Significance vs. controls: ** p<0.01, *** p<0.001; Significance vs. acute CRPS: ^++^ p<0.01, ^+++^ p<0.001; Significance vs. ipsilateral hand: ^(§)^ p<0.10, ^§^ p<0.05, ^§§§^ p<0.001. Note: Significance marks that bridge symbols of ipsilateral and contralateral hands apply to both hands. Error bars show 1SEM.

ANCOVA with age as a covariate (partialing out the highly significant age-related effects) revealed a highly significant effect of group (F_2,113_ = 30.04, p<<0.0001), body side (ipsilateral vs. contralateral: F_1,113_ = 12.39, p<0.001) and group x side interaction (F_2,113_ = 4.65, p<0.05) for the cold detection threshold (CDT). In healthy control subjects, CDT was highly correlated (r = 0.74, p<0.0001) and very symmetrical between body sides (mean CDT: 1.27 vs. 1.26°C; p = 0.88) (Table 3). In contrast, CDT was significantly less correlated between body sides in CRPS patients (r = 0.43, p<0.001; difference of correlation vs. controls: p<0.02). CDT was significantly increased in acute CRPS patients (2.25°C; p<<0.0001 vs. controls) and even more increased in chronic CRPS (3.66°C; p<<0.0001 vs. controls and p<0.001 vs. acute CRPS) (Figure 2A). CDT in the contralateral hand of acute CRPS patients was significantly lower than in the ipsilateral hand, and although marginally increased (1.45°C), it did not differ from healthy controls (p = 0.26). In contrast, in chronic CRPS, CDT was considerably increased in the contralateral hand with only a marginal difference to the ipsilateral hand (2.85°C, p<<0.0001 vs. controls and acute CRPS; p<0.05 vs. affected hand) (Figure 2A). Thus, patients with acute CRPS displayed pronounced ipsilateral cold hypoaesthesia. In chronic CRPS, this phenomenon was more pronounced and also found in the contralateral hand.

**Table 3:** Thermal detection in acute and chronic CRPS.

*Group*	*Acute CRPS*		*Chronic CRPS*		*Control*	
Examined Hand	Ipsilateral	Contralateral	Ipsilateral	Contralateral	Dominant	Contralateral
**CDT** Mean (Mean log±SD)	2.25°C (0.353±0.377)	1.45°C (0.160±0.258)	3.66°C (0.564±0.338)	2.85°C (0.455±0.257)	1.27°C (0.106±0.249)	1.26°C (0.100±0.253)
**WDT** Mean (Mean log±SD)	4.48°C (0.651±0.337)	2.87°C (0.457±0.263)	5.18°C (0.714±0.297)	4.16°C (0.619±0.247)	2.16°C (0.334±0.222)	2.19°C (0.340±0.226)
**TSL** Mean (Mean log±SD)	7.47°C (0.873±0.378)	4.59°C (0.662±0.281)	9.00°C (0.954±0.288)	6.49°C (0.812±0.211)	3.30°C (0.518±0.269)	3.56°C (0.551±0.260)

Cold Detection Threshold

WDT: Warm Detection Threshold

TSL: Thermal Sensory Limen

Warm detection thresholds (WDT) exhibited a similar overall pattern: ANCOVA (group: F_2,113_ = 27.66, p<<0.0001, body side: F_1,113_ = 13.12, p<0.001, and group x side interaction: F_2,113_ = 4.22, p<0.05), loss of symmetry (2.16°C vs. 2.19°C; p = 0.88 in healthy controls), and deterioration of correlation between sides (r = 0.56 vs. r = 0.35) (Table 3). WDT was increased in the ipsilateral hand of acute and chronic CRPS patients (4.48°C and 5.18°C; p<0.001 each vs. controls) (Figure 2B). Furthermore, WDT was significantly increased in the contralateral hand of acute and chronic CRPS (2.87°C and 4.16°C; p<0.01 and p<<0.0001 vs. controls) (Figure 2B). In aggregate, there was warm detection hypoaesthesia in the ipsilateral and contralateral hands of acute or chronic CRPS patients with no significant difference between hands in the chronic CRPS group.

Thermal sensory limen (TSL), assessed by alternating CDT and WDT, yielded the same results: ANCOVA (group: F_2,113_ = 22.42, p<<0.0001, body side: F_1,113_ = 19.12, p<0.001 and group x side interaction: F_2,113_ = 10.36, p<0.0001) revealed a strong ipsilateral threshold increase in acute CRPS, even more increased in chronic CRPS (7.47°C and 9.00°C vs. 3.30°C in controls; p<<0.0001 each). A similar pattern was observed in the contralateral hand (4.59°C and 6.49°C; p<<0.0001, each vs. controls, and p<0.005, each vs. the affected hand) (Table 3).

### Thermal Pain Thresholds

**Figure 3:**
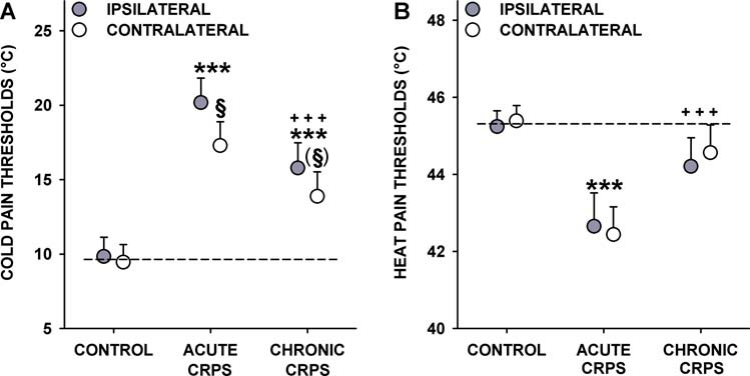
Thermal pain thresholds in acute and chronic CRPS. Cold pain thresholds (A) and heat pain thresholds (B) are significantly lowered in acute CRPS (corresponding to cold and heat hyperalgesia). Pain partially (cold pain) or totally (heat pain) recovers to normal in chronic CRPS. Significant increases of similar magnitude are also found in the contralateral “unaffected” hand for heat pain (B) and to a lesser extent in cold pain (A). Significance vs. controls: *** p<0.001; Significance vs. acute CRPS: ^+++^ p<0.001; Significance vs. ipsilateral hand: ^(§)^ p<0.10, ^§^ p<0.05. Note: Significance marks that bridge symbols of ipsilateral and contralateral hands apply to both hands. Error bars show 1SEM.

A very different pattern was found for thermal pain thresholds. Heat pain thresholds (HPT) exhibited a highly significant group effect in ANCOVA (F_2,113_ = 7.33, p<0.001), but neither body side, nor group x side interaction were significant (both p>0.70). HPT was symmetrical in all groups (p>0.50 each) and significantly correlated between body sides although significantly less well in CRPS (r = 0.52 vs. r = 0.77, p<0.05). HPT was significantly lowered in acute CRPS patients when compared to healthy controls (42.65±0.87°C vs. 45.24±0.41°C, p<0.001). In chronic CRPS, however, HPT had almost normal values (44.21±0.74°C, p>0.10 vs. controls, but p<0.001 vs. acute CRPS; Figure 3B, Table 4). Thus, acute CRPS patients displayed a bilateral heat hyperalgesia, almost absent in chronic CRPS.

**Table 4:** Thermal nociception in acute and chronic CRPS.

*Group*	*Acute CRPS*		*Chronic CRPS*		*Control*	
*Examined Hand*	*Ipsilateral*	*Contralateral*	*Ipsilateral*	*Contralateral*	*Dominant*	*Contralateral*
**HPT** Mean±SD	42.65±0.87°C	42.44±0.72°C	44.21±0.74°C	44.56±0.72°C	45.24±0.41°C	45.39±0.40°C
**CPT** Mean±SD	20.18±1.64°C	17.29±1.60°C	15.79±1.69°C	13.88±1.64°C	9.83±1.30°C	9.45±1.19°C

HPT: Heat Pain Thresholds

CPT: Cold Pain Threshold

Likewise, cold pain thresholds (CPT) exhibited a highly significant effect of group (F_2,113_ = 11.84, p<0.0001) and body side (F_1,113_ = 7.75, p<0.01) in ANCOVA, but no group x side interaction (p = 0.22). High symmetry (mean CPT: 9.83±1.30 vs. 9.45±1.19°C; p = 0.65) and correlation in healthy subjects was not found in CRPS patients (r = 0.70 vs. r = 0.85) (p<0.05 vs. controls:) (Table 4). Acute CRPS patients exhibited bilateral cold hyperalgesia (CPT: 20.18±1.64°C ipsilaterally and 17.29±1.60°C contralaterally, p<<0.0001 each vs. controls; side-to-side comparison p<0.05). In chronic CRPS, when compared to acute CRPS, cold hyperalgesia was significantly less pronounced tending towards normal thresholds (15.79±1.69°C in the ipsilateral and 13.88±1.64°C in the contralateral hand; p<<0.0001 each vs. acute CRPS). However, CPT remained significantly different from controls (p<0.0001 in both hands) (Figure 3A).

### A Standardised View of Thermal Sensitivity in CRPS Patients

**Figure 4:**
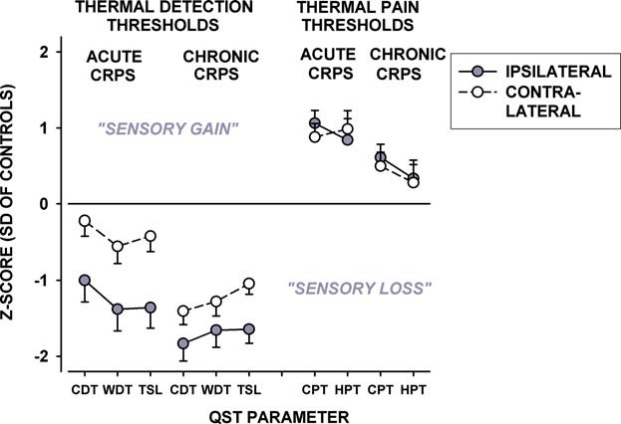
Standardised comparison of QST data normalised to mean and standard deviation of the control group (z-normalisation). Normalised data show a severe sensory loss in acute CRPS for all thermal detection parameters (CDT, WDT, TSL) in the affected ipsilateral hand (>1 SD of controls), but also a moderate loss in the contralateral hand, which aggravates in chronic CRPS. In contrast, there is a substantial gain in thermal nociception (CPT, HPT) in acute CRPS (hyperalgesia), equally expressed in both hands (≈1 SD of controls). Heat hyperalgesia almost fully subsides in chronic CRPS, while significant cold hyperalgesia is retained. For the sake of clarity there are no symbols of statistical significance in this figure (c.f. respective paragraph in results).

The pattern of changes in different parameters of thermal sensitivity were compared in normalized data (z- transformed vs. control subjects) [21] (Figure 4). ANCOVA on normalized QST data correcting for the significant age-dependency of all QST parameters (Rao's R = 8.26 for covariate age, p<<0.0001) revealed significant main effects of group, side, thermal detection vs. thermal pain (all p<0.001), but not of thermal modality (hot vs. cold, p = 0.25). Notably, group x side interaction was weak and failed to reach significance (F_2,113_ = 2.37, p = 0.07), indicating that overall side differences of sensitivity in CRPS patients were not marked. In contrast, a highly significant interaction for group x thermal detection vs. thermal pain was found (F_2,113_ = 46.62, p<<0.0001) based on pronounced sensory loss (thermal hypoaesthesia) relative to the control group in acute CRPS, and even more pronounced in the chronic stage. In contrast, a gain (hyperalgesia) in thermal pain (CPT, HPT) was found, which was less marked in the chronic stage of CRPS (Figure 4). A highly significant interaction of side x thermal detection vs. pain (F_1,113_ = 23.28, p<<0.0001) was based on more prominent sensory loss in thermal detection in the affected hand of CRPS patients. Separate ANCOVAs for thermal detection or pain thresholds of CRPS patients confirmed a significant asymmetry of loss in thermal detection (affected >> contralateral; F_1,113_ = 26.28, p<0.0001), but not in thermal pain (F_1,113_ = 0.22, p = 0.63).

### Paradoxical Heat Sensations

**Figure 5:**
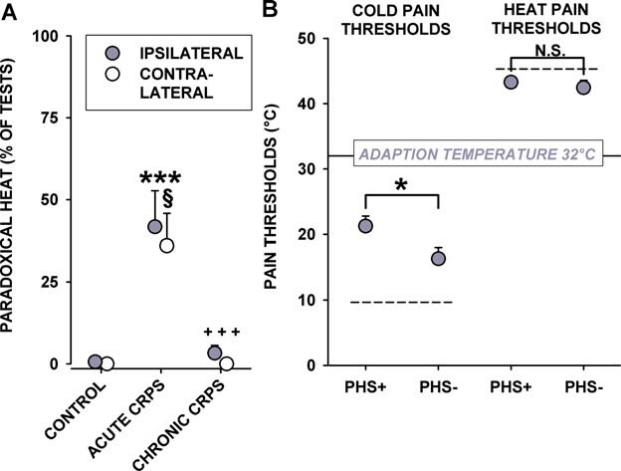
(A) Paradoxical heat sensation (PHS) to mild cold stimuli as elicited by alternating cold and warm stimulation (TSL). PHS was a frequent finding in both hands in acute CRPS, and fully subsided in the chronic phase. Significance vs. controls: *** p<0.001; Significance vs. acute CRPS: ^+++^ p<0.001; Significance vs. ipsilateral hand: ^(§)^ p<0.10, ^§^ p<0.05. Note: Significance marks that bridge symbols of ipsilateral and contralateral hands apply to both hands. (B) PHS-positive CRPS patients (PHS+) exhibited a significantly more pronounced cold hyperalgesia than PHS-negative CRPS patients (PHS−). In contrast, there was no such difference in heat hyperalgesia. Error bars show 1SEM.

Paradoxical heat sensations (PHS) occurred in only 1/336 TSL trials in both hands of healthy controls (0.3%). In contrast, PHS was a very frequent finding in the affected and contralateral hand in acute CRPS (9/20 patients and 25/60 tests = 41.7% and 9/25 patients and 27/75 tests = 36%, p<<0.0001 each vs. controls; p<0.05 affected vs. contralateral hand; Spearman R rank correlation = 0.87 between both hands; Figure 5A). In contrast, PHS was almost completely absent in chronic CRPS and only encountered in 3/93 tests ipsilaterally (3.2%) in 2/31 patients and 0/99 tests contralaterally (0/33 patients; both p<<0.0001 vs. acute CRPS).

In further analyses, the impact of the presence (PHS+) or absence (PHS−) of PHS on any other sensory measure was explored. ANCOVAs (corrected for age) revealed no impact of PHS+ or PHS− on either CDT, WDT, TSL or HPT (all p>0.40). In contrast, PHS+ and PHS– patients differed significantly in their cold pain thresholds (ANCOVA: F_1,42_ = 7.36, p<0.01). PHS+ patients displayed significantly more cold pain hyperalgesia than PHS– patients (mean CPT: 21.24±1.55°C vs. 16.31±1.73°C, p<0.05), but no difference in e.g. heat hyperalgesia (mean HPT: 43.28±0.75°C vs. 42.44±1.11°C, p = 0.47; Figure 5B).

Stepwise multiple regression analysis identified three variables that significantly predicted the occurrence of PHS (multiple regression R = 0.55, p<0.002), namely age (partial r = 0.44, p<0.005), duration of acute CRPS (partial r = 0.35, p<0.05), and CPT (partial r = 0.39, p<0.02).

## Discussion

As there is a vivid discussion about the pathophysiological mechanisms underlying CRPS I and II [10], [12], [14], there are still only few publications providing quantitative information about the sensory changes occurring in this neuropathic pain disorder. Despite the fact that sensory changes are a “conditio sine qua non” for the diagnosis of a CRPS [1], [2], the character, degree and chronological order of those changes are relatively unexplored, and therefore, the pathomechanisms remain cryptic. The studies hitherto applying Quantitative Sensory Testing (QST) for patients with CRPS revealed inconsistent or even partially conflicting results. Thimineur and colleagues were the first to describe sensory changes contralateral to the affected side. However, they could not define a distinct thermal sensory profile concerning the affected limb [4]. The largest number of CRPS-patients characterised by a structured QST protocol examined 57 patients, respectively. This study demonstrated warm hypoaesthesia as well as cold hyperalgesia in the affected limb [19]. Eisenberg and colleagues tested a small sample of 12 patients with CRPS of either the upper or lower extremity and reported significant changes in heat and cold pain thresholds, but not in the detection thresholds for warm and cold [27]. None of these groups were able to detect changes in the contralateral limb. Paradoxical heat sensation was not tested in either of these studies. It may be noted, that all indexed studies substantially differ in their applied QST protocols. Recently, Üçeyler and colleagues were the first to apply a standardised protocol, as suggested by the German Research Network on Neuropathic Pain (DFNS) including thermal testing, PHS and mechanical testing [28]. In their study, 32 patients suffering from CRPS I or II were examined by means of QST, showing significant thermal hypoesthesia in the diseased limb.

In the study at issue, a standardised protocol for thermal testing was used [21]. Patients suffering from CRPS I of the upper extremity were included in the study, exclusively. No patients with CRPS II or CRPS of the lower extremities were enclosed, as the probable pathophysiology of CRPS I differs from CRPS II, and the sensory thresholds of the upper extremity significantly differ from those on the lower extremity [20]. The precise assessment of the time since inciting event enabled to define the sensory changes occurring at different stages of the disease. Furthermore, an age and gender matched control group was tested, in order to reveal differences in sensory profiles in CRPS patients compared to healthy subjects.

### Distinction Between the Acute and Chronic CRPS Group

The distinction of acute vs. chronic CRPS was justified by a natural gap in an obviously bimodal distribution concerning duration of disease (Figure 1). Furthermore, patients in the acute group displayed more pronounced clinical signs of inflammation, as signalled by the occurrence of edema and a positive side to side difference in skin temperature (Table 2). This preponderance of inflammatory signs in acute CRPS is in accordance with the literature [7], [29], [30]. In the chronic CRPS group, a considerably lesser occurrence of edema was registered, and concomitantly, the overall side to side difference in skin temperature was negative.

### Possible Pathomechanisms of Acute CRPS

#### Inflammation

Besides pain, acute CRPS is inter alia accompanied by edema, reddening and increased skin temperature. Any of these symptoms (tumor, calor, rubor) suggest the presence of inflammation, and thus it has been hypothesized, that at least in the early phase, aseptic inflammation is a critical feature of CRPS [7]. As neurogenic mechanisms strongly contribute to aseptic inflammation [31], neurogenic inflammation might be a possible starting point of the inflammatory process in CRPS. This is underlined by experimental findings of increased levels of calcitonine gene-related peptide (CGRP) and facilitated neurogenic inflammation in acute CRPS [10], [32].

In this study, the presence of heat hyperalgesia in the acute CRPS group indicates peripheral sensitisation of heat-sensitive C-fibre nociceptors, which is a hallmark sign of inflammatory processes [33]. The simultaneous presence of cold hyperalgesia can also be explained by peripheral sensitisation, as Wasner and colleagues have shown that cold hyperalgesia in humans is likely mediated by sensitisation of cold-sensitive C-nociceptors [34]. The existence of hyperalgesia for noxious heat and cold stimuli in acute CRPS strongly supports the hypothesis of an inflammatory process [35].

### Small Fibre Degeneration

Apart from signs of inflammatory hyperalgesia, our findings suggest a pronounced degeneration of thinly myelinated A-delta cold fibres as well as unmyelinated C warm fibres in acute CRPS. The degeneration hypothesis is based on a highly significant increase in warm and cold detection thresholds, indicating emerging A-delta and C-fibre dysfunction [22], [36], [37] (Figure 2). This degenerative process might be triggered by the ongoing aseptic inflammation.

Apart from inflammation, the presence of cold hyperalgesia can also be explained by a second mechanism. This involves central disinhibition of cold-sensitive nociceptive pathways, namely by an degenerative insufficiency of A-fibre inputs to control nociceptive inputs, since cold hyperalgesia is reliable induced by experimental acute and selective A-fibre conduction blockade [34]. Wasner and colleagues interpreted this effect as a lack of C-fibre inhibition normally exerted by a concomitant activation of cold sensitive A-delta fibres.

The hypothesis of cold pain hyperalgesia being caused by a combination of inflammation and A-delta-fibre degeneration is emphasised by the incomplete recovery of CPT in chronic CRPS. Heat hyperalgesia, which is mainly caused by inflammatory C-fibre sensitisation, resolved almost completely, as suggested by a return of HPT to almost normal values in the chronic stage of the disease. At the same time, significant cold hyperalgesia persisted in the chronic stage, when signs of axonal degeneration were even more pronounced (see Figure 3).

### Synergism of Inflammation and Small Fibre Degeneration in Acute CRPS

Paradoxical heat sensation (PHS), i.e. a sensation of hot or burning pain to mild cold stimulation following a preceding mild warm stimulus, represents a disturbance of sensory integration in thermosensation. It is hardly ever present in the hands of healthy subjects (never in females of any age, and only at a rate of 0.6% in males >40 years of age) [20]. In contrast, in the study at issue, the prevalence of PHS in acute CRPS patients was unusually high (approximately 40%). Two mechanisms contribute to the appearance of PHS, either disinhibition of a heat-sensitive C-fiber pathway by blockade, or loss of A-fibre input and facilitation of this already disinhibited pathway by sensitisation of the respective primary afferents [23], [38]. The presence of PHS fosters the hypothesis of a synergism of inflammation and small fibre degeneration as being the two major pathomechanisms acting in acute CRPS. Thus, in PHS, the disturbance of sensory integration is hypothesised to be based on A-fibre loss. This is supported by the strong increase in cold detection threshold, suggesting an at least partially dysfunctional cold-sensing A-delta fibre pathway, which in turn disinhibits the cold-sensitive polymodal nociceptive C-fibre pathway. The C-fibre pathway was boosted by inflammatory sensitisation, thus further increasing the likelihood of PHS. This is in accordance with the finding, that cold hyperalgesia was partially resolved in chronic CRPS, but remained significantly present at a lesser level, although the inflammatory hyperalgesia was fully resolved, as signified by the absence of heat hyperalgesia. Notably, PHS did not occur anymore in chronic CRPS (see Figure 5A). The positive correlation between cold pain hyperalgesia and the occurrence of PHS in acute CRPS additionally confirms the hypothesis, that cold pain hyperalgesia, just like PHS, is caused by A-delta fibre degeneration as well as inflammation (Figure 5B). Moreover, the correlation between CPT and HPT leads to the suggestion, that inflammatory hyperalgesia (indicated by the lowering of HPT) also had an impact on cold pain sensitivity and hence indirectly on the presence of PHS.

### Possible Pathomechanisms of Chronic CRPS

#### Progressing Small Fibre Degeneration

In chronic CRPS, symptoms of inflammation disappear, while neurological signs of small nerve fibre degeneration prevail. As discussed above, paradoxical heat sensation not only requires an A-delta fibre dysfunction, but is also more likely to occur when C-fibre input is increased, as in the condition of inflammatory peripheral sensitisation. In chronic CRPS, when clinical signs of inflammation subside, PHS was thus almost absent. Furthermore, no heat hyperalgesia was detectable, indicating the absence of a relevant peripheral inflammation. Additionally, cold as well as warm detection thresholds deteriorated, indicating a further impairment of small fibre function. This is in order with a recent study by Oaklander and colleagues, showing that CRPS I leads to small fibre axonal degeneration [12]. Interestingly, of the 18 patients participating in their study, only two had a history of disease shorter than one year, so most of their patients met our criteria for chronic CRPS.

### Contralateral Sensory Changes

Unexpectedly, in acute as well as in chronic CRPS I, QST-results revealed changes in the contralateral hand, which mirrored the sensory changes in the hand primarily affected by the disease. These changes were not always as pronounced as the alterations on the ipsilateral (affected) side. Namely, there was a lesser degree of loss of cold and warm detection. Notably however, hyperalgesia in acute CRPS was equally pronounced on the ipsilateral as well as on the contralateral side. This might necessitate the conclusion, that inflammatory hyperalgesia is not limited to the affected limb. This may be related to an exaggerated level of neurogenic inflammation, since an increased axon reflex vasodilation has been shown to occur in the contralateral hand of former CRPS patients [39]. We suggest that neurogenic inflammation might be a predisposing factor for the development of this disease. A bilateral interaction has been demonstrated for neurogenic inflammation, which is accompanied by a segmentally organised short-lived suppression of this inflammatory response in the innervation territory of the respective contralateral nerve, mediated by somatostatin release [40]. Such bilateral interaction has remained unexplained and even been ignored for a long time. In recent publications, however, there is evidence from animal models as well as from clinical studies, that both suspected pathomechanisms, neurogenic inflammation as well as small fibre degeneration, show a bilateral distribution in unilateral animal models of chronic joint pain (e.g. monoarthritis) or in neuropathic pain disorders, like unilateral nerve injury and postherpetic neuralgia [41]–[43]. Conversely, evidence of small fibre degeneration in the innervation territory contralateral to the extremity affected by CRPS could not be demonstrated [12]. Furthermore, consistent with our results, Coderre and colleagues were able to detect a contralateral spread of hyperalgesia in an animal model of acute CRPS [5]. These experimental findings are supported by clinical case reports, which show contralateral sensory changes in other neuropathic pain syndromes like trigeminal neuralgia [44]. Likely, contralateral changes frequently remain undetected, because they are often unincisive, and many studies lack an adequate control group.

### Limitations of the Current Study

QST is a behavioural functional measure of sensory function. Thus, it does not provide direct evidence of either structural loss of axons innervating the tested area or phenotypic changes of sensory nerve fibers.

So far, epidermal nerve fibre density and Quantitative Sensory Testing show only weak correlations [45]. Although intra-individual correlation is not warranted, however, sensory loss as revealed by QST and axonal loss run in parallel as a group result [46]–[48].

In the absence of structural changes, an increase in thermal detection thresholds can also result from functional impairment of a sensory pathway. Particularly, tactile but also thermal sensitivity is modulated dynamically by nociceptive input, and hypoaesthesia secondary to clinical as well as experimental pain conditions has been delineated [49]–[51]. As tactile hypoaesthesia in those experimental protocols seems to be related to the degree of central (i.e. spinal or cortical) plasticity [52], the thermal hypoaesthesia found in this study might be partially due to central inhibition of non-noxious thermal input. However, the magnitude of hypoaesthesia observed in our patients was more pronounced than it would be expected from studies on pain induced hypoaesthesia [50], [52]. Therefore, it is unlikely that this type of central plasticity can account for this degree of changes alone.

Furthermore, the involvement of supraspinal changes must be taken into account, particularly in order to understand the contralateral spread of symptoms. This is important, since thalamic as well as cortical reorganisation has been demonstrated to occur in patients suffering from CRPS I [14], [53]. The sensory changes observed in our patient cohort might well represent the origin of cortical reorganisation in CRPS I, as central reorganisation can be mediated peripherally [54].

As a note of caution to the interpretation of our data, however, the degree of cortical reorganisation in the respective studies seemed to be associated with an impairment of tactile discrimination and was well correlated with the extent of mechanical hyperalgesia [14], [16], [55]. Neither of these parameters nor a test for mechanical allodynia was included in our test protocol. The latter would also be capable to estimate the degree of central sensitisation and hence maybe related to central reorganisation [56]. Therefore, estimating the relative importance of central changes may be more closely targeted by studies including a more comprehensive protocol of sensory testing. Finally, a longitudinal prospective follow-up study would enable to confirm the results of this study and to identify possible “risk-profiles” for the development of a chronic CRPS.

In conclusion, the present QST results suggest that in acute CRPS I, aseptic neurogenic inflammation accompanies or even initiates tissue changes, precipitating peripheral sensitisation and thus leading to hyperalgesia. Concomitantly, the same fundamental process may also initiate a hitherto unknown sequence of responses that eventually lead to the degeneration of small fibres. The exact time course of these events is not delineated yet. However, in chronic CRPS I, inflammation and ensuing signs of peripheral hyperalgesia subside, while at the same time, the degeneration of A-delta and C-fibres might further progress. It is unknown, whether this represents a final stage of trophic changes. All QST changes were, to a lesser degree, also present in the contralateral limb, indicating that pathophysiological changes in CRPS I might also be subclinically present in the extremity which is not primarily affected by the disease.
